# Evaluation of Targeted Influenza Vaccination Strategies via Population Modeling

**DOI:** 10.1371/journal.pone.0012777

**Published:** 2010-09-17

**Authors:** John Glasser, Denis Taneri, Zhilan Feng, Jen-Hsiang Chuang, Peet Tüll, William Thompson, Mary Mason McCauley, James Alexander

**Affiliations:** 1 National Center for Immunization and Respiratory Diseases, Centers for Disease Control and Prevention, Atlanta, Georgia, United States of America; 2 Department of Mathematics, Purdue University, West Lafayette, Indiana, United States of America; 3 Epidemic Intelligence Center, Centers for Disease Control, Taipei, Taiwan; 4 Scientific Advice Unit, European Centre for Disease Prevention and Control, Stockholm, Sweden; University of Minnesota, United States of America

## Abstract

**Background:**

Because they can generate comparable predictions, mathematical models are ideal tools for evaluating alternative drug or vaccine allocation strategies. To remain credible, however, results must be consistent. Authors of a recent assessment of possible influenza vaccination strategies conclude that older children, adolescents, and young adults are the optimal targets, no matter the objective, and argue for vaccinating them. Authors of two earlier studies concluded, respectively, that optimal targets depend on objectives and cautioned against changing policy. Which should we believe?

**Methods and Findings:**

In matrices whose elements are contacts between persons by age, the main diagonal always predominates, reflecting contacts between contemporaries. Indirect effects (e.g., impacts of vaccinating one group on morbidity or mortality in others) result from off-diagonal elements. Mixing matrices based on periods in proximity with others have greater sub- and super-diagonals, reflecting contacts between parents and children, and other off-diagonal elements (reflecting, e.g., age-independent contacts among co-workers), than those based on face-to-face conversations. To assess the impact of targeted vaccination, we used a time-usage study's mixing matrix and allowed vaccine efficacy to vary with age. And we derived mortality rates either by dividing observed deaths attributed to pneumonia and influenza by average annual cases from a demographically-realistic SEIRS model or by multiplying those rates by ratios of (versus adding to them differences between) pandemic and pre-pandemic mortalities.

**Conclusions:**

In our simulations, vaccinating older children, adolescents, and young adults averts the most cases, but vaccinating either younger children and older adults or young adults averts the most deaths, depending on the age distribution of mortality. These results are consistent with those of the earlier studies.

## Introduction

Seasonal influenza causes an estimated 200,000 hospitalizations and 36,000 deaths on average in the United States, most among the elderly [1]. If a 1918-like pandemic occurred today, 10 million hospitalizations and 1.9 million deaths – many among younger adults – are expected [2]. Vaccination affords the best protection, especially for those at risk of pneumonia and other life-threatening complications [1].

Development and production of influenza vaccines is challenging. In the northern hemisphere, the World Health Organization (WHO) collects relevant information every February for review by experts. Based on which viruses they believe will most likely be circulating, the experts select 3 strains for inclusion in the upcoming season's vaccine. Almost every year, at least one vaccine constituent is replaced, because viral strains drift; i.e., undergo constant genetic change. Even small changes can result in novel strains, and mismatch with circulating strains can reduce vaccine effectiveness, as occurred during the 2007–08 influenza season [3]. Once the experts have identified the strains likely to circulate next season, a vaccine must be manufactured in a slow process that has changed little since its invention. Testing, approval, and distribution also take several months. Problems encountered during production, such as inability to grow sufficient quantities of a viral strain, may cause vaccine shortages or delays in distribution. Such problems have affected vaccine availability in recent influenza seasons in the United States [4].

During influenza pandemics, these challenges are compounded. Pandemic strains may emerge when antigenic shifts – major changes in the genetic makeup of a virus – occur in influenza A, creating new viral subtypes against which populations have little or no immunity [5]. Even when effective vaccines are created, acute shortages are possible, especially in areas with limited production capacity that also have little advance warning, making it difficult or impossible to obtain sufficient vaccine in time to protect at-risk populations. During the recent pandemic, even in wealthy countries that developed and produced an H1N1 vaccine as soon as possible, vaccine supplies were inadequate to accommodate all who sought timely vaccination. The prospect of a shortage motivated health authorities in influenza vaccine-producing countries to devise strategies for ensuring that people who were most likely to suffer complications of influenza were vaccinated first. In the United States, the CDC's Advisory Committee on Immunization Practices (ACIP) determined that pregnant women, caregivers of young infants, health care workers, and people too young to have antibodies to H1N1 had first priority. Next were those most vulnerable to complications of influenza, generally the elderly [6].

In such circumstances, other strategies for using scarce influenza vaccine efficiently also warrant consideration. Among such strategies is indirect protection; that is, immunizing those who might infect vulnerable people. One group whose vaccination might achieve the benefits of indirect protection is schoolchildren. The merits of vaccinating schoolchildren against influenza, partly to protect others, such as the elderly, have been argued from community-intervention trials [7], natural experiments [8], and individual-based models [9]. While trials generally are better controlled than natural experiments, they are relatively expensive and time-consuming. Moreover, only models allow examination of alternative vaccination strategies in exactly the same setting. Models should be evaluated against historical observations to check their predictive ability, but identifying and remedying deficiencies of individual-based models can be prohibitively difficult. Population models are simple enough for evaluation before use to inform public policy making. Analytical results, such as the optimal targets for interventions against infectious diseases, also can be derived.

To identify vaccine allocation strategies with the greatest potential to reduce influenza morbidity and mortality, we studied an age-structured population model whose infection rates we estimated from observed proportions infected [10] and interpersonal contacts weighted by duration [11]. Our model's disease-induced mortality rates were either quotients of deaths attributed to pneumonia or influenza [12] and populations at risk or products of those rates and ratios of 1918 and average 1913–17 mortalities [13]. We refer to the latter as contemporary 1918-like mortality.

## Methods

We adapted a demographically-realistic version of a classic population model [14] with 4 disease or immune states: susceptible; infected, but not yet infectious (exposed); infectious; recovered and immune (removed). We added vaccination with age-specific efficacy, based on the work of Govaert et al., who conducted the only randomized, double-blind, placebo-controlled trial of vaccination against morbidity [15], and loss of immunity to circulating strains, via antigenic shifting and drifting [16]. For lack of the requisite information, we ignored transient protection via maternal antibodies, despite how important this protection may be, given seasonal influenza complications among infants aged <6 months [17]. For simplicity, we also ignored immigration and emigration. File S1 and Table S1 describe the system of equations and parameter values.

Age-structured models require multiple infection rates, to which Anderson and May [18] referred collectively as “who-acquires-infection-from-whom.” We derived ours from age-specific proportions of household members infected during the 1957 influenza pandemic [10], commonly called “attack rates,” and from interpersonal contacts weighted by duration in Portland, Oregon [11]. Briefly, the risks of infection, 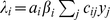, where *a_i_* are average numbers of contacts per person per day; *β_i_* are probabilities of infection upon contact with infectious persons; *c_ij_* are proportions of contacts that members of group *i* have with those of group *j*; and *y_j_* = *I_j_*/*N_j_* are probabilities that randomly encountered members of group *j* are infectious. Using a logistic regression model fitted to the *y_i_* reported by Chin et al. [10] and the duration-weighted contacts of Del Valle et al. [11], together with the relationships 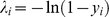 and 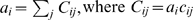, we estimated *λ_i_* and then *β_i_*.

**Figure 1:**
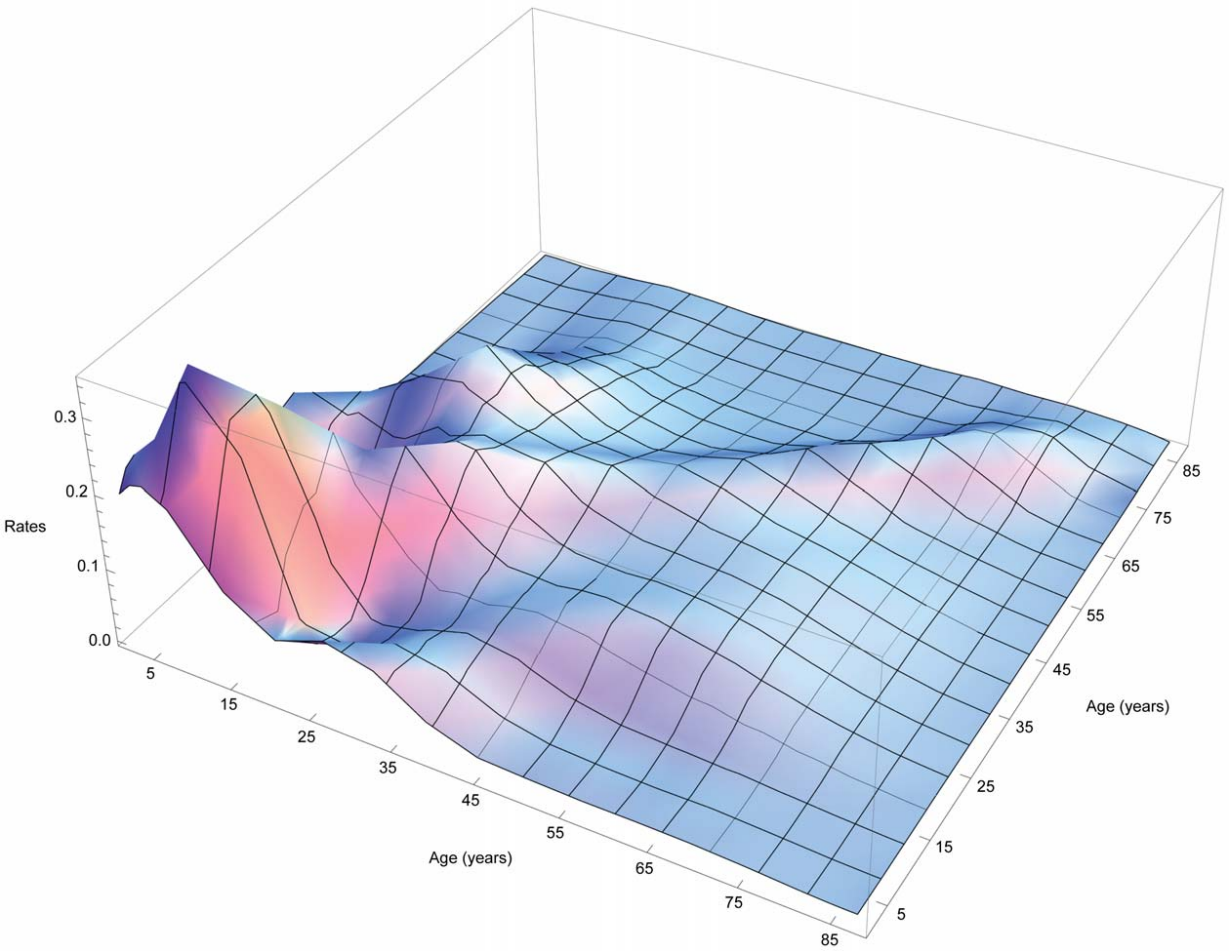
Effective contact or infection rates derived from attack “rates” during the 1957 pandemic [10] and daily contacts weighted by duration [11].

Figure 7 of Glasser et al. [unpublished manuscript] illustrates *β_i_* obtained using these “attack rates” and the *C_ij_* from several recently published studies of face-to-face conversations or periods in proximity with others during which respiratory diseases might be transmitted. Del Valle et al. [11] not only weighted contacts by duration, but the off-diagonal elements of their contact matrix are relatively large, increasing possible indirect effects (e.g., impact of vaccination on morbidity or mortality in groups not targeted), and they kindly shared their observations. Thus, we could calculate empirical rates of effective contact between members of any age groups simply by averaging (Figure 1).

**Figure 2:**
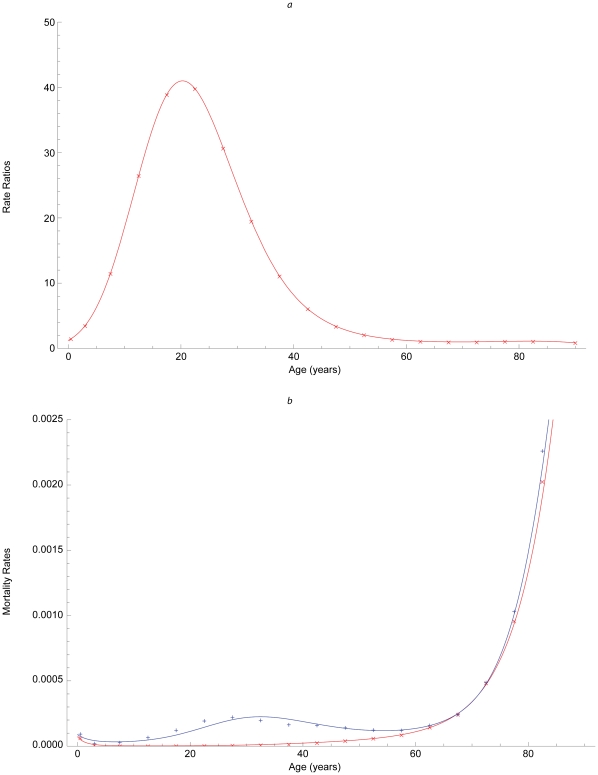
Mortality due to influenza. (a) Ratios of pandemic (1918) and pre-pandemic (1913–1917) mortality rates [13]; (b) rates derived from deaths attributed to pneumonia and influenza in the United States during 2005 (red), and their product with the ratios above (blue).

We calculated age-specific disease-induced mortalities as quotients of 2005 deaths attributed to pneumonia or influenza (Table S1) and simulated infections during an average year. We obtained contemporary 1918-like mortalities by fitting logistic regression models to published 1918 and average 1913–17 rates [13], calculating age-specific ratios for the groups we modeled, <1, 1–4, 5–9, …, 80–84, 85+ years (Figure 2a), and multiplying them by the 2005 rates. Our estimates of mortality conditional on influenza during 2005 and a hypothetical contemporary 1918-like pandemic are illustrated in Figure 2b.

In experiments, all else should be equal. We simulated our model without vaccination, with 60% of infants <1 year and adults ≥65 years of age or the same percentage of children aged 1–9, adolescents 10–19, or young adults 20–29 years being vaccinated. These groups are roughly the same size, but coverage actually is <60% among persons <65 years of age [19]. Our hypothetical annual influenza vaccine protected 70% of people 1–64 years of age, but lower proportions of infants and older adults. Efficacy was 35% among infants and declined linearly with age over 64 years (i.e., was 60% among people aged 65–69 years, 50% among those aged 70–74 years, and so on). The resulting efficacies among elderly adults correspond roughly to those reported by Govaert et al. [15]. Annual vaccination occurred November through January; pandemic vaccination began 30 days later and continued for 6 months. Pandemic efficacy was half annual, but as roughly twice as many doses were eventually administered, similar numbers of people were protected.

To assess the impact of these alternative strategies on morbidity and mortality, we averaged daily differences between age-specific cases or deaths with and without vaccination over 365-day periods. Averaging was necessary because our simulation model is stochastic (i.e., we employ Renshaw's discrete event/time method [20]). Finally, in age-structured models, the average number of effective contacts, ℜ_0_, may be calculated as the dominant eigenvalue of the next-generation matrix [21] whose associated eigenvector describes the age-specific contributions [22], [23]. We derive these quantities in File S2.

## Results

Our matrix of infection rates (Figure 1) illustrates preferential mixing, not only among contemporaries – which is particularly intense among older children, adolescents, and young adults [24] – but also that between parents and children and among co-workers evident in more recent, higher-resolution observations [11], [25], [26]. As indirect effects emanate from off-diagonal matrix elements, such observations increase the accuracy of assessments of intervention impacts via transmission modeling. Similarly, our 1918-like mortalities (Figure 2) resemble those of the 2009 pandemic, although this swine H1N1 was much less virulent than that avian strain.

**Figure 3:**
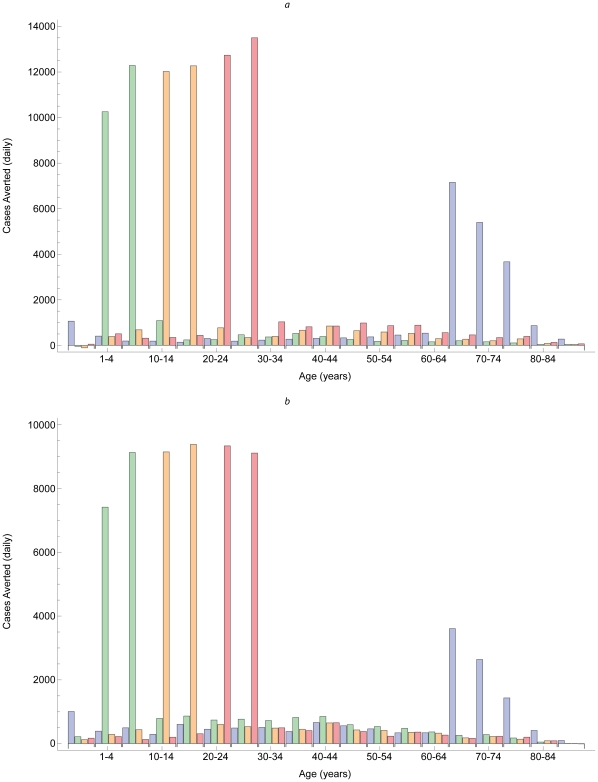
Cases averted by vaccination. Similar patterns in cases averted by vaccinating people aged <1 year and 65+ years (blue bars), 1–9 years (green bars), 10–19 years (yellow bars), and 20–29 years (red bars) during hypothetical annual (a) and pandemic (b) outbreaks.

**Figure 4:**
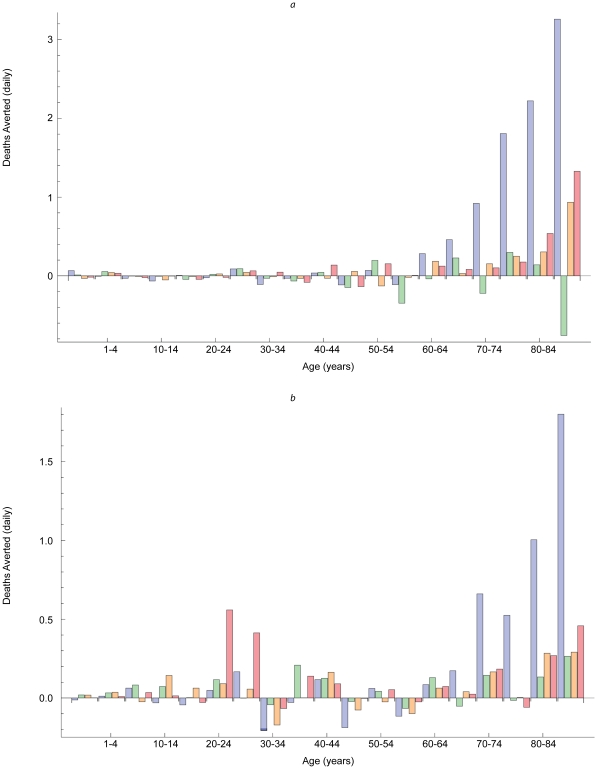
Deaths averted by vaccination. Dissimilar patterns in deaths averted by vaccinating people aged <1 year and 65+ years (blue bars), 1–9 years (green bars), 10–19 years (yellow bars), and 20–29 years (red bars) during hypothetical annual (a) and pandemic (b) outbreaks.

During simulated pandemic as well as annual influenza outbreaks, vaccinating older children, adolescents, and young adults reduced morbidity the most, especially among target age groups (Figures 3a and b). Despite a contact matrix with relatively large off-diagonal elements, only 20–25% of cases averted were in groups not targeted. By contrast, vaccinating infants and elderly adults reduced mortality most during simulated annual influenza outbreaks (Figure 4a), but vaccinating young adults also reduced mortality during simulated pandemics (Figure 4b).

**Figure 5:**
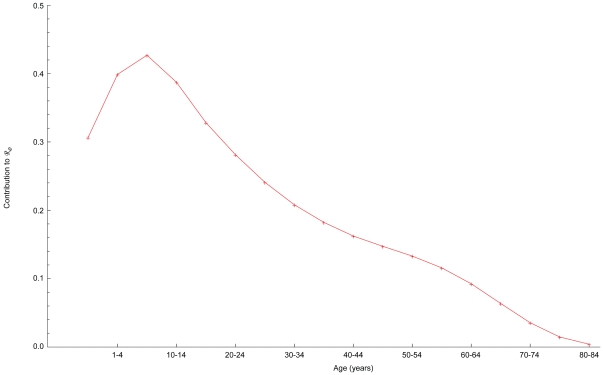
Normalized age-specific contributions to the reproduction number (File S2).

While target age groups are similar in size, the numbers of cases averted depend on the vaccine efficacies as well as age distribution of the 2005 U.S. population. Cases averted per efficacy adjusted dose correspond to the proportionate contributions to ℜ_0_ (Figure 5), which identifies the optimal target for interventions to reduce transmission.

## Discussion

We adapted a classic age-structured population model with parameters chosen to maximize indirect effects due to vaccinating older children, adolescents, and young adults, and to accurately assess direct effects due to vaccinating elderly adults. Comparing the impact of vaccinating these age groups against influenza, we found that vaccinating children, adolescents, and young adults would reduce morbidity the most, with 20–25% of the reduction in other age groups. However, while vaccinating infants and older adults would mitigate mortality most during annual outbreaks, vaccinating young adults also would mitigate mortality during contemporary 1918-like pandemics.

Evidently, which vaccination strategy is superior depends on the objective: mitigating morbidity or mortality, and if mortality, its age-distribution. For many years, U.S. vaccination policy was designed to mitigate mortality, particularly among elderly adults. Relatively recently, it was redesigned to also mitigate morbidity, initially among young children, but then progressively among older children, adolescents, and adults [http://www.cdc.gov/media/pressrel/2010/r100224.htm]. Unlike this policy, in which the 6 month lower age of recommended vaccination has not changed as the upper age has increased, our experimental design maintained similar target group sizes by increasing both lower and upper ages of vaccination simultaneously.

Our findings are comparable to those obtained via other methodologies. The observation that mortality attributed to influenza and pneumonia among elderly Japanese was lower when children were vaccinated routinely [8] suggests that susceptible young people pose a risk to elderly ones, but not necessarily directly. While few such studies are unequivocal, numerous U.S. experiences [27] are consistent with this deduction. Similar conclusions have been reached via community intervention trials [28], [29], [30] as well as individual-based modeling [9]. As our findings support results of these studies using other methodologies, they make a strong case for using relatively simple population models to examine pressing public health issues, and therefore to arrive relatively quickly at sound conclusions about the effectiveness of alternative interventions.

Influenza vaccination strategies have been compared recently using a variety of modeling approaches and perspectives. In 2007, Dushoff et al. [31] explored the same strategies in a 2-group model, one more effective at transmitting the pathogen and other more vulnerable to its effects. These researchers were reluctant to choose among the many interesting scenarios described by various combinations of their parameters, and urged only caution. In 2006, Bansal et al. [32] adopted a more detailed network model with which they also evaluated these strategies, obtaining results qualitatively similar to ours. Three years later, Medlock and Galvani [33] used an age-structured population model with a mixing matrix whose off-diagonal elements are relatively small [25]. Nonetheless, they concluded that vaccinating older children, adolescents, and young adults was the best strategy, regardless of objective.

Impacts of other control measures for pandemic influenza also have been explored recently, by modeling individual members of socially and spatially structured populations [34]–[38]. Our work illustrates several advantages of simpler population models [39]. Insofar as plausible mixing scenarios are modeled, individual behavior is extraneous. Furthermore, systems of equations can be analyzed, whereas computer programs cannot; for example, Areno et al. [40] not only reproduced results with a proportionately-mixed, age-structured population model that had been obtained with a relatively complex individual-based model [9], but also deduced several analytical results. Finally, population models use observations and make predictions familiar to epidemiologists, who group individuals based on characteristics of interest, both in disease surveillance, and to develop and implement interventions. As recently as 2008, for example, Vynnycky and Edmunds used a population model to investigate the impact of school closures on the spread of influenza during a pandemic [41].

Because people of some ages are more active than others, immunizing those potential “super-spreaders” reduces the average number of secondary infections disproportionately. As Figure 5 indicates, adolescents and young adults are the optimal targets for reducing morbidity. Because the main diagonal predominates in all known mixing matrices [11], [23], [25], [26], however, direct effects exceed indirect ones. Unless vaccine efficacy is very low, consequently, the best strategy for reducing mortality will be to vaccinate members of at-risk groups [42]. This analytical result is not limited to vaccination; it may be applied to other interventions that prevent infection or reduce the magnitude or duration of infectiousness. For example, as neuraminidase inhibitors are most effective when administered early [43], timely medication of ill children, adolescents, and young adults could reduce the number needing treatment and possibly the duration of treatments. Treating optimally would be much less costly than widespread prophylaxis, and reduce the risk of drug-resistant strains emerging [44].

Age-specific infection rates are the essence of population models. We calculated risks of infection from Chin et al.'s prospective study of household transmission following illnesses among schoolchildren [10]; households without school-aged children were not represented. Together with clinical observations and individual onset dates, a cross-sectional serological survey would remedy this possible deficiency and might resolve uncertainty about the contribution of asymptomatic infections to transmission. Anderson and May [18] described “who-acquires-infection-from-whom” matrices with as many unique elements as risks of infection, but Nold [45] formulated mixing as a convex combination of age-specific activities (number of contacts per person per day) and constant preference (proportion with others in the same group), and Jacquez et al. [46] allowed preference to vary with age. Recent empirical observations enabled us to include contacts between parents and children and among co-workers [Glasser et al. unpublished manuscript]. Insofar as mixing differs from society to society, if not between rural and urban sub-populations, more diverse subjects would permit continued refinement of methods to permit rapid, robust analysis and interpretation of alternative actions to address public health priorities.

## Supporting Information

File S1 A classic population model.
(0.02 MB DOC)

File S2 The reproduction number.
(0.06 MB DOC)

File S3 Evaluation of the model.
(0.02 MB DOC)

Table S1 Demographic parameters, United States, 2005 [12].
(0.07 MB DOC)
