# Supplementary material for: Evaluation of Targeted Influenza Vaccination Strategies via Population Modeling
Source: PLoS One. 2010 Sep 17;5(9):e12777. doi: 10.1371/journal.pone.0012777 (PMC2941445; doi:10.1371/journal.pone.0012777)
Supplement: File S1 — A classic population model. (0.02 MB DOC) [file pone.0012777.s001.doc]

*I. A Classic Population Model*

Our model population is composed of susceptible, exposed (infected, but not yet infectious), infectious, and removed (recovered and immune) individuals who, by virtue of antigenic drifting or shifting, lose immunity *to circulating pathogens*.

Molecular epidemiology suggests that antigenic shifts occur via recombination at 5-year intervals on average, between which clusters of more closely related strains arise via mutation [16], whereupon ω = (5×365.25 days)^-1^. Births and deaths occur at per capita rates, *μ* = (75×365.25 days)^-1^, which amounts to ignoring disease-induced mortality in the analyses that follow. Our simulation model includes such mortality, at rates calculated as described in the main text, and is otherwise realistic demographically (i.e., births and deaths attributed to causes other than influenza occur at rates that are either tabulated or obtainable from information in Table S1, respectively). We also ignore passively-acquired maternal antibodies and immigration/emigration. With subscripts denoting age groups, the resulting system of equations is:

$$\begin{matrix} \frac{dS_{i}}{dt}=\mu N_{i}+\omega R_{i}-\left(\lambda_{i}+\mu\right)S_{i} \\ \frac{dE_{i}}{dt}=\lambda_{i}S_{i}-\left(\alpha+\mu\right)E_{i} \\ \frac{dI_{i}}{dt}=\alpha E_{i}-\left(\gamma+\mu\right)I_{i} \\ \frac{dR_{i}}{dt}=\gamma I_{i}-\left(\omega+\mu\right)R_{i} \\ \text{where }\lambda_{i}=a_{i}\beta_{i}\sum_{j} c_{ij}\left(\frac{I_{j}}{N_{j}}\right)=a_{i}\beta_{i}\sum_{j} c_{ij}y_{j}, \\ \text{and} N_{i}=S_{i}+E_{i}+I_{i}+R_{i}. \end{matrix}$$

In *λ_i_*, the risks of infection per susceptible person in age group *i*, the *a_i_* are average numbers of contacts per person per day, the *β_i_* are probabilities of infection upon contact with infectious people, the *c_ij_* are proportions of their contacts that members of group *i* have with members of group *j*, and the *y_j_* = *I_j_*/*N_j_* are probabilities that randomly encountered members of group *j* are infectious. Probabilities from Chin et al. [10] and *C_ij_* = *a_i_c_ij_* from Del Valle et al. [11] enable us to calculate the *β_i_* via the definition of *λ_i_* in the main text. The remaining per capita rates, *α* and *γ*, are reciprocals of the latent and infectious periods, 1^-1^ and 3.8^-1^ days, respectively. These values are based on the observation that healthy volunteers shed virus for 4.8 days on average, with amounts increasing during the first day post-infection [47].

**REFERENCE**

1. Carrat F, Vergu E, Ferguson NM, Lemaitre M, Cauchemez S, et al. (2008) Time lines of infection and disease in human influenza: a review of volunteer challenge studies. Am J Epidemiol 167:775-785.
