# Supplementary material for: Evaluation of Targeted Influenza Vaccination Strategies via Population Modeling
Source: PLoS One. 2010 Sep 17;5(9):e12777. doi: 10.1371/journal.pone.0012777 (PMC2941445; doi:10.1371/journal.pone.0012777)
Supplement: File S2 — The reproduction number. (0.06 MB DOC) [file pone.0012777.s002.doc]

*II. The Reproduction Number*

**Case I.** Age class *i* is isolated: $\text{ }\lambda_{i}=a_{i}\beta_{i}\left(\frac{I_{i}}{N_{i}}\right)$.

In this case, we may obtain the reproduction number for group *i* via either the endemic or disease-free equilibrium. The endemic equilibrium$\text{ }V_{i}^{*}=(u_{i}^{*},x_{i}^{*},y_{i}^{*},z_{i}^{*})$ can be solved as:

$u_{i}^{*}=\frac{1}{\Re_{0i}},\text{ }x_{i}^{*}=\left(1-\frac{1}{\Re_{0i}}\right)\left[1+\frac{\alpha}{\alpha+\mu}\left(1+\frac{\gamma}{\omega+\mu}\right)\right],\text{ }y_{i}^{*}=\frac{\alpha}{\alpha+\mu}x_{i}^{*},\text{ }z_{i}^{*}=\frac{\alpha}{\alpha+\mu}\frac{\gamma}{\omega+\mu}x_{i}^{*}$,

where $\Re_{0i}=\frac{\alpha a_{i}\beta_{i}}{(\gamma+\mu)(\alpha+\mu)}$.

Alternatively, consider the disease-free equilibrium. In this case, the next generation matrix is:

$K_{1}=\left(\begin{matrix} \frac{\alpha a_{1}\beta_{1}}{(\alpha+\mu)(\gamma+\mu)} & \frac{a_{1}\beta_{1}}{\gamma+\mu} & 0 & 0 & \cdots& 0 & 0 \\ 0 & 0 & 0 & 0 & \cdots& 0 & 0 \\ 0 & 0 & \frac{\alpha a_{2}\beta_{2}}{(\alpha+\mu)(\gamma+\mu)} & \frac{a_{2}\beta_{2}}{\gamma+\mu} & \cdots& 0 & 0 \\ 0 & 0 & 0 & 0 & \cdots& 0 & 0 \\ \vdots& \vdots& \vdots& \vdots& \vdots& \vdots& \vdots\\ 0 & 0 & 0 & 0 & \cdots& \frac{\alpha a_{n}\beta_{n}}{(\alpha+\mu)(\gamma+\mu)} & \frac{a_{n}\beta_{n}}{\gamma+\mu} \\ 0 & 0 & 0 & 0 & \cdots& 0 & 0 \end{matrix}\right)$.

Because groups are isolated, this is a diagonal matrix. The non-zero elements, $\Re_{0i}=\frac{\alpha a_{i}\beta_{i}}{(\gamma+\mu)(\alpha+\mu)},$are the sub-population reproduction numbers. The meta-population reproduction number is the dominant eigenvalue of *K*_1_, which is $\Re_{0}=\max\{\Re_{01},\Re_{02},\cdots,\Re_{0n}\}.$

**Case II**. Age classes are not isolated:$\lambda_{i}=a_{i}\beta_{i}\sum_{j} c_{ij}\left(\frac{I_{j}}{N_{j}}\right)$.

In this case, the endemic equilibrium$\text{ }V_{i}^{*}=(u_{i}^{*},x_{i}^{*},y_{i}^{*},z_{i}^{*})$ generally cannot be solved explicitly. The component $y_{i}^{*}$ satisfies the following system of quadratic equations:

$\Re_{0i}\left[1-\left(1+\frac{\gamma}{\omega+\mu}+\frac{(\alpha+\mu)(\gamma+\mu)}{\alpha}\right)y_{i}^{*}\right]\sum_{j} c_{ij}y_{j}^{*}=y_{i}^{*},\text{ }i=1,2,\cdots n$.

Now we consider the disease-free equilibrium. In this case, the next generation matrix is:

$$K_{2}=\left(\begin{matrix} \frac{\alpha a_{1}\beta_{1}c_{11}}{(\alpha+\mu)(\gamma+\mu)} & \frac{a_{1}\beta_{1}c_{11}}{\gamma+\mu} & \frac{\alpha a_{1}\beta_{1}c_{12}}{(\alpha+\mu)(\gamma+\mu)} & \frac{a_{1}\beta_{1}c_{12}}{\gamma+\mu} & \cdots& \frac{\alpha a_{1}\beta_{1}c_{1n}}{(\alpha+\mu)(\gamma+\mu)} & \frac{a_{1}\beta_{1}c_{1n}}{\gamma+\mu} \\ 0 & 0 & 0 & 0 & \cdots& 0 & 0 \\ \frac{\alpha a_{2}\beta_{2}c_{21}}{(\alpha+\mu)(\gamma+\mu)} & \frac{a_{2}\beta_{2}c_{21}}{\gamma+\mu} & \frac{\alpha a_{2}\beta_{2}c_{22}}{(\alpha+\mu)(\gamma+\mu)} & \frac{a_{2}\beta_{2}c_{22}}{\gamma+\mu} & \cdots& \frac{\alpha a_{2}\beta_{2}c_{2n}}{(\alpha+\mu)(\gamma+\mu)} & \frac{a_{2}\beta_{2}c_{2n}}{\gamma+\mu} \\ 0 & 0 & 0 & 0 & \cdots& 0 & 0 \\ \vdots& \vdots& \vdots& \vdots& \vdots& \vdots& \vdots\\ \frac{\alpha a_{n}\beta_{n}c_{n1}}{(\alpha+\mu)(\gamma+\mu)} & \frac{a_{n}\beta_{n}c_{n1}}{\gamma+\mu} & \frac{\alpha a_{n}\beta_{n}c_{n2}}{(\alpha+\mu)(\gamma+\mu)} & \frac{a_{n}\beta_{n}c_{n2}}{\gamma+\mu} & \cdots& \frac{\alpha a_{n}\beta_{n}c_{nn}}{(\alpha+\mu)(\gamma+\mu)} & \frac{a_{n}\beta_{n}c_{nn}}{\gamma+\mu} \\ 0 & 0 & 0 & 0 & \cdots& 0 & 0 \end{matrix}\right)$$

$\Re_{0}$ is the dominant eigenvalue of the matrix *K*_2_. Note that this matrix has *n* zero eigenvalues. The others are given by an *n* by *n* matrix,

$$K_{3}=\left(\begin{matrix} \Re_{01}c_{11} & \ldots& \Re_{01}c_{1n} \\ \vdots& \ddots& \vdots\\ \Re_{0n}c_{n1} & \cdots& \Re_{0n}c_{nn} \end{matrix}\right),$$

which highlights the importance of mixing. Because *K_3_* is a positive matrix, its dominant eigenvalue must be positive. The probabilities of transmission upon contact with infectious people in our model reflect differences in susceptibility. If these probabilities also or only reflected differences in infectiousness, the off-diagonal elements would be of the form $\frac{\alpha a_{i}\beta_{ij}c_{ij}}{(\alpha+\mu)(\gamma+\mu)},$ which cannot be written as ℜ_0i_ × *c_ij_*. While one cannot generally obtain explicit formulae for dominant eigenvalues when *n*>3, one can always calculate them numerically. When *n*=2, for example, the dominant eigenvalue of matrix *K_3_* is
