# Supplementary material for: Evaluation of Targeted Influenza Vaccination Strategies via Population Modeling
Source: PLoS One. 2010 Sep 17;5(9):e12777. doi: 10.1371/journal.pone.0012777 (PMC2941445; doi:10.1371/journal.pone.0012777)
Supplement: File S3 — Evaluation of the model. (0.02 MB DOC) [file pone.0012777.s003.doc]

*III. Evaluation*

We evaluated this model’s ability to reproduce hospitalizations and mortality due to influenza in Taiwan, estimating only age-specific coefficients of harmonic functions by which the version used for this purpose was seasonally forced and probabilities of hospitalization conditional on influenza. The coefficient of variation of predictions and hospitalizations during the period of 2000-08, based on claims to Taiwan’s National Health Insurance program, was only ≈ 30%, but the probability of hospitalization given influenza was about 0.0005 overall, in agreement with independent observations (Table 15, Health Utilization Statistics, Ministry of Health, Taiwan), the age-specific estimates were reasonable (i.e., j-shaped), and the proportion of P&I mortality due to influenza was strikingly similar to the 9.8% reported by Thompson et al. [48]. Clearly multiple strain or type models would better reproduce such observations, but – as our goal was to develop a simple model that could be used to evaluate various age-targeted vaccination strategies – this complexity seemed unwarranted. These and other results of our modeling of influenza in Taiwan are available from the first author on request.

**REFERENCE**

1. Thompson WW, Shay DK, Weintraub E, Brammer L, Cox N, et al. (2003) Mortality associated with influenza and respiratory syncytial virus in the United States. JAMA 289:179-186.
