# Supplementary material for: Evaluation of Targeted Influenza Vaccination Strategies via Population Modeling
Source: PLoS One. 2010 Sep 17;5(9):e12777. doi: 10.1371/journal.pone.0012777 (PMC2941445; doi:10.1371/journal.pone.0012777)
Supplement: Table S1 — Demographic parameters, United States, 2005 [12]. (0.07 MB DOC) [file pone.0012777.s004.doc]

Table S1. Demographic parameters, United States, 2005 [12]. In the simulation modeling, mortality rates due to other causes were obtained by dividing the difference between all cause and P&I deaths by the population and due to influenza by dividing P&I deaths by average influenza infections during several 365-day simulations.

| Age (years) | Population | Pr(female) | Birth Rates | Deaths | P&I Deaths |
| --- | --- | --- | --- | --- | --- |
| <1 | 4,104,279 | 0.488563 |  | 27,940 | 267 |
| 1–4 | 16,210,858 | 0.488746 |  | 4,786 | 110 |
| 5–9 | 19,557,631 | 0.488533 |  | 2,888 | 51 |
| 10–14 | 20,878,743 | 0.487863 | 0.000660 | 3,947 | 55 |
| 15–19 | 21,063,122 | 0.487088 | 0.040410 | 13,708 | 68 |
| 20–24 | 21,052,873 | 0.484246 | 0.102051 | 19,718 | 104 |
| 25–29 | 20,054,422 | 0.489437 | 0.115288 | 20,419 | 147 |
| 30–34 | 20,090,234 | 0.494911 | 0.095615 | 20,455 | 207 |
| 35–39 | 21,005,651 | 0.497575 | 0.046227 | 40,882 | 318 |
| 40–44 | 22,860,332 | 0.502759 | 0.009107 | 44,492 | 616 |
| 45–49 | 22,485,966 | 0.506268 | 0.000538 | 94,058 | 955 |
| 50–54 | 20,001,365 | 0.510623 | 0.000041 | 83,665 | 1,228 |
| 55–59 | 17,352,661 | 0.514622 |  | 151,340 | 1,522 |
| 60–64 | 13,001,917 | 0.523101 |  | 113,395 | 1,900 |
| 65–69 | 10,133,719 | 0.533996 |  | 217,195 | 2,486 |
| 70–74 | 8,516,293 | 0.552456 |  | 182,529 | 4,137 |
| 75–79 | 7,418,928 | 0.579390 |  | 388,752 | 7,123 |
| 80–84 | 5,640,765 | 0.617040 |  | 295,576 | 11,440 |
| 85+ | 5,077,302 | 0.686063 |  | 671,870 | 30,267 |
| Total | 296,507,061 |  |  | 2,397,615 | 63,001 |
